# Pioglitazone improves skeletal muscle functions in reserpine-induced fibromyalgia rat model

**DOI:** 10.1080/07853890.2021.1916069

**Published:** 2021-07-07

**Authors:** Fatma E. Hassan, Hader I. Sakr, Passant M. Mohie, Howayda Saeed Suliman, Ayman Saber Mohamed, Mohamed H. Attia, Dalia M. Eid

**Affiliations:** a Department of Medical Physiology, Faculty of Medicine, Cairo University, Egypt; b Department of Medical Physiology, Batterjee Medical College, Jeddah, Saudi Arabia; c Department of Clinical Pharmacology, Faculty of Medicine, Alexandria University, Egypt; d Department of Medical Biochemistry, Faculty of Medicine, Alexandria University, Egypt; e Department of Zoology, Faculty of Science, Cairo University, Egypt; f Internal Medicine Department, Faculty of Medicine, Ain Shams University, Egypt; g Department of Biochemistry, Faculty of Medicine, Ain Shams University, Egypt

**Keywords:** Fibromyalgia, PPAR- γ, catalase, superoxide dismutase, malondialdehyde, IL-8

## Abstract

**Background:**

Fibromyalgia (FM) is characterized by musculoskeletal pain, fatigue, sleep and memory disturbance. There is no definitive cure yet for FM-related health problems. Peroxisome proliferator-activated receptor’s (PPAR’s) activation is associated with insulin sensitisation and improved glucose metabolism. PPAR-γ was reported to alleviate FM allodynia. Limited data are discussing its effect on motor disorders.

**Objective:**

To investigate the potential effect of PPAR-γ agonists (pioglitazone, as one member of thiazolidinediones (TZD)) on motor dysfunction in reserpine-induced FM in a rat model.

**Materials and methods:**

Thirty-six male Wistar rats were divided into negative control (*n* = 9) and reserpine-induced FM (*n* = 27) groups. The latter was subdivided into three equal subgroups (*n* = 9), positive control (untreated FM model), pioglitazone-treated and GW9662-treated. We evaluated muscle functions and activity of chloramphenicol acetyltransferase, superoxide dismutase, malondialdehyde, and serum levels of interleukin-8 and monocyte chemoattractant protein-1.

**Results:**

Pioglitazone significantly relieved fatigue, improved muscle performance, reduced inflammatory cytokines and enhanced antioxidant’s activity, while GW9662, a known PPAR-γ antagonist, aggravated the FM manifestations in the rat model.

**Conclusion:**

PPAR-γ agonists show a promising role against FM-associated motor dysfunctions.

## Introduction

Fibromyalgia (FM) is an example of a chronic-pain health problem that draws the attention of many health care practices worldwide [1]. FM is characterized by diffuse pain, body tenderness to pressure stimuli and morning stiffness, which progressively lead to increased fatigue, sleep disorders and psychological instabilities in many patients [2,3]. The underlying pathophysiologic mechanisms of FM are not clear. There are some reports of the multisystem failure process involving the immune, musculoskeletal and central nervous systems (CNS) [4,5]. Some authors believe that FM is a familial disorder, a hypothesis supported by a few lines of evidence correlating certain single nucleotide polymorphisms (SNPs) in genes involved in neurotransmitter synthesis (e.g. serotonin, dopamine and catecholamine), endocrinal disturbances of the hypothalamic-pituitary-adrenal axis, or increased levels of substance P (mediating the pain control system) to the development of FM [6].

Despite the multidisciplinary management regimens used for FM patients, there is no definitive cure for this chronic health problem [7]. Therefore, it is important to have a better understanding of the pathophysiology underlying FM to develop a more effective therapy and/or improve the current treatment modalities, which have shown only limited success compared with their serious side effects [8].

To develop better treatments for fibromyalgia symptoms, we had to implement an animal model which mimics the features of fibromyalgia patients. Repeated administration of reserpine (1 mg/kg subcutaneous, once daily, for three consecutive days) causes a significant decrease in tactile allodynia and the muscle pressure threshold, both in male and female rats. This is accompanied by decreased amounts of biogenic amines (dopamine, norepinephrine and 5-hydroxytryptamine) in the prefrontal cortex, thalamus and the spinal cord, which are deeply involved in pain signal processing. It also significantly increases immobility time in the forced swim test as indicative of depression, which is a common comorbid symptom of fibromyalgia. This animal model provides face validity (manifestation of chronic pain and comorbid symptoms), construct validity (dysfunction of biogenic amine-mediated CNS pain control is involved) and predictive validity (similar responses to treatments used in fibromyalgia patients) [9].

Peroxisome proliferator-activated receptor (PPAR) -γ agonists are insulin sensitizers used for the treatment of type II diabetes [10]. They have a promising analgesic synergy with other chronic-pain medications [11] by reducing allodynia and hyperalgesia in neuropathic pain when given intrathecal [12] or systemically [13–15].

Thus, we hypothesize that PPAR-γ agonists, through counteracting hyperalgesia and allodynia, would improve skeletal muscle function and mitigate fatigue in an FM-rat model. Our study will investigate the effect of PPAR-γ agonists on skeletal muscle functions in reserpine-induced fibromyalgia in the male Wistar rat model. We will also study PPAR-γ agonist's effects on cellular oxidative stress and inflammatory cytokines production.

## Materials and methods

### Animal care and housing

Thirty-six adult male Wistar rats, weighing 215 (±15 g), were quarantined at the animal house of Kasr Al Aini Faculty of Medicine, Cairo University, for 15 days before the start of experimentation for adaptation and to exclude for any signs of infection. Rats were housed in plastic cages (three rats/cage) with well-aerated covers under standard temperature conditions (25 ± 5 °C) and 12-hours alternating light and dark cycles. Rats were given free water access and supplied daily with a laboratory rat diet offered ad-lib. The described protocols in this study were approved by the Institutional Animal Care and Use Committee (CU-IACUC), Cairo University, with approval number: CU/I/F/66/20.

### Animal grouping

Rats were randomly divided into two main groups:

1. The negative control (group I): A total of nine rats received subcutaneous (SC) injections of distilled water (1 mg/kg) for three consecutive days and then were injected with 1% dimethyl sulfoxide (DMSO) solution intraperitoneal (IP) for a total period of 21 days.
2. Fibromyalgia group (group II): A total of 27 rats were SC injected with reserpine (1 mg/kg diluted in 0.5% glacial acetic acid (acetic acid in distilled water) once daily. Injections were made into variable sites of the rat’s flanks for three consecutive days to create a model that mimics FM [16]. Rats with FM signs were then divided randomly into three equal subgroups (*n* = 9 each):
  - The positive control (subgroup IIa): Rats received an IP injection of 1% DMSO solution for 21 days.
  - The pioglitazone (subgroup IIb): Rats received IP injection of 1% DMSO and pioglitazone (>99% purity) 15 mg/kg by gavage, daily for 21 days [17].
  - The GW9662 (subgroup IIc): Rats received GW9662 (1.5 mg/kg dissolved in 0.1% DMSO by IP injection once daily for 21 days [18].

### Chemicals

1. Pioglitazone was purchased from Amoun pharmaceuticals, Egypt.
2. Reserpine was purchased from Novartis pharmaceuticals, Egypt.
3. DMSO solution, acetic acid, GW9662 and phenobarbital was purchased from Sigma Aldrich (St. Louis, MO) and Millipore (Billerica, MA).

### Animal sacrifice and sample collection

At the end of the experimental period, animals were sacrificed by the carbon-di-oxide (CO_2_) euthanasia method [19]. Blood samples were collected from jugular veins. Blood was centrifuged at 3000 rpm for 15 min. Using a clean pipette technique, clear non-haemolysed sera were aliquoted into three 1.5 ml Eppendorf tubes for each rat and stored at −20^°^ C [20].

### *Skeletal muscle functions* (using a computer system and power lab 26 T)

The proximal tendon of the extensor digitorum longus (EDL) muscle was dissected from the femoral compartment of the hind limb. The anterior crural compartment was exposed by dissecting the biceps femoris. The distal tendons of EDL were dissected out intact from the foot through distal fasciotomy, with the removal of connective tissue and ligaments. The entire EDL muscle was mounted on a 25ml organ bath system (Harvard Apparatus, Holliston, MA), containing Krebs-Ringer bicarbonate buffer that was continuously bubbled with a mixture of 95% oxygen (O_2_) and 5% CO_2_, and kept at 30 °C. The four distal EDL tendons were tied together by non-absorbable surgical silk suture and fixed to a support. The proximal tendon was tied to the force transducer [21], the latter of which was connected to iWorx advanced animal/human physiology data acquisition unit AHK/214 (Harvard Apparatus).

Muscles were stimulated to supramaximal levels by platinum electrodes placed directly on muscles parallel to their longitudinal axes. The length of muscles was adjusted via a micromanipulator to reach maximum isometric-twitch tension using 1 Hz electrical stimulations with a 1 min rest period in between. Time-to-peak twitch tension and time taken by muscles to relax to 50% of their peak twitch tensions were recorded.

To obtain maximum fused-tetanic tension, muscles were evoked at different frequencies (10, 30, 50, 70, 90 and 110 Hz) for 1 s, followed by 3 min rest after each stimulation and responses were recorded. The optimal frequency at which maximum fused tension was reached was recorded and used for subsequent experimentation.

Next, we assessed time-to-fatigue and muscle recovery from fatigue. Muscles were stimulated at the optimal frequency, reached in the previous step, for 1 second, followed by 5 s rest for a total of 5 min. The muscle response was recorded every minute after initial maximum fused tension and expressed as a decline in force from maximum fused tension. Time to weakest-possible response was considered time-to-fatigue. To estimate recovery from fatigue, muscles were allowed to rest for 5 min, and tetanic tension was recorded thereafter. Normalized muscle tensions were recorded and expressed as Newton per gram (N/g) wet muscle mass [22].

### *Measurement of serum levels of monocyte chemoattractant protein* (*MCP)-1 and interleukin (IL)-8*

Serum levels of MCP and IL-8 were done by ELISA assays MyBioSource company, Catalog # MBS2709256 and # MBS9141543, respectively, as previously described [23].

### Measurement of skeletal muscle homogenate oxidants-antioxidants markers

Chloramphenicol acetyltransferase (CAT) activity was measured using Aebi's methods [24] (CAT assay kit, Catalog # MBS9718961; MyBioSource, San Diego, CA). Superoxide dismutase activity was measured using a spectrophotometric methodology as described by Oyanagui [25] (SOD assay kit, Catalog # MBS168803; MyBioSource). Malondialdehyde (MDA) concentration was measured by Rat MDA ELISA Kit, Catalog # MBS9712310, MyBioSource and calculated from the standard curve, prepared from 1,1,3,3-tetra ethoxy propane [26].

### Statistical analysis

Data were coded and entered using Windows statistical software package SPSS 15.0 (SPSS Inc., Chicago, IL). Data were summarized using mean and standard deviation for quantitative variables. Analysis of variance with multiple comparisons *post hoc* test was done for statistically significant differences among the mean value of different groups [27]. *p* Values less than.05 was considered statistically significant.

## Results

### Skeletal muscle functions

#### Maximum isometric-twitch tension, time-to-peak twitch tension and time-to-relax to 50%

**Chart 1.**
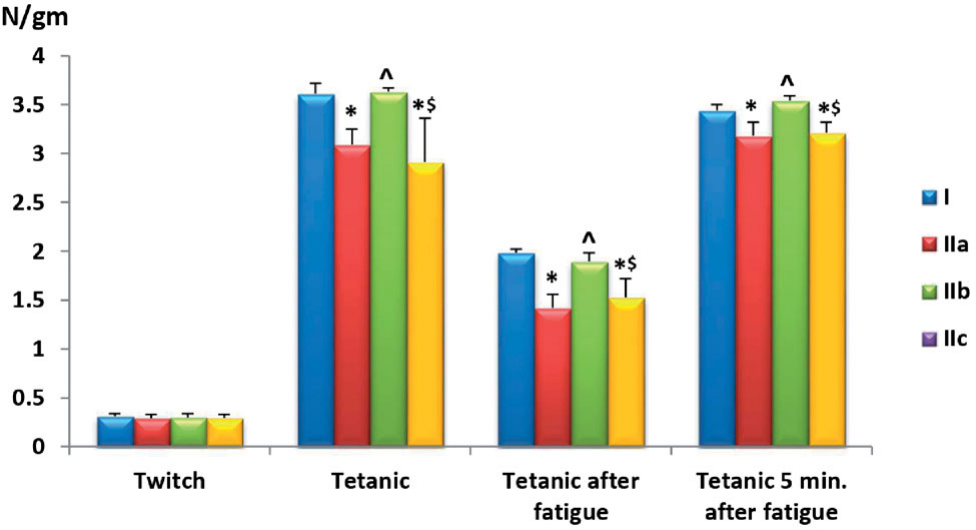
Comparison of maximum twitch tension, maximum tetanic tension, maximum tetanic tension after fatigue and maximum tetanic tension 5 min following fatigue among the study groups. Values are presented as mean ± SD. Statistically significant (*p* < .05) as compared to the corresponding value in *group I, **^**group IIa and ^$^group IIb.

**Chart 2.**
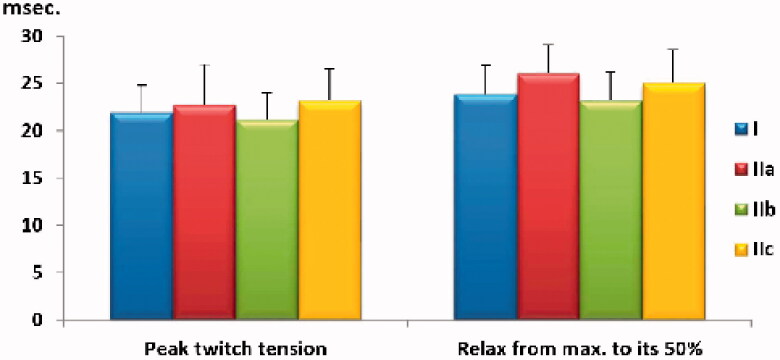
Comparison of time for peak twitch tension and time to relax from maximum tension to 50% of its value among the study groups. Values are presented as mean ± SD.

As shown in Table 1, we found no statistically significant differences (*p* > .05) in any of the fibromyalgia groups compared to the negative control group regarding maximum isometric-twitch and time-to-peak twitch tensions, neither in time-to-relax from maximum twitch tension to their 50% values (Chart 1). No statistically significant (*p* > .05) differences in the aforementioned parameters were noted when comparing pioglitazone- or GW9662-treated groups to the positive control group, or when comparing pioglitazone to GW9662 treatment (Chart 2).

**Table 1:** . Representation of different parameters among the study groups.^a^

Group		I	II		
			a	b	c
Twitch	Max. tension (N/gm)	0.31 ± 0.03	0.29 ± 0.04	0.3 ± 0.04	0.291 ± 0.04
Tetanic		3.61 ± 0.11	3.09 ± 0.16*	3.63 ± 0.04^	2.91 ± 0.45*^$^
Tetanic after fatigue		1.98 ± 0.04	1.42 ± 0.14*	1.89 ± 0.09^	1.53 ± 0.19*^$^
Tetanic 5 min after fatigue		3.44 ± 0.06	3.18 ± 0.14*	3.54 ± 0.05^	3.21 ± 0.11*^$^
Peak twitch tension	Time (msec)	21.93 ± 2.88	22.68 ± 4.29	21.17 ± 2.79	23.14 ± 3.37
Relax from maximum to its 50%		23.82 ± 3.09	26.04 ± 2.997	23.14 ± 3.06	25.03 ± 3.5
IL-8	Pg/ml	529.5 ± 20.4	781.88 ± 64.98*	609.8 ± 53.86*^	818.13 ± 31.99*^$^
MCP-1		792.5 ± 27.86	1199.4 ± 50.43*	503.6 ± 23.77*^	1302.2 ± 48.77*^^$^
CAT (IU/mg)		63.85 ± 9.5	29.31 ± 2.18*	59.85 ± 3.09^	36.17 ± 3.84*^$^
SOD (NU/mg)		92.39 ± 3.99	80.6 ± 2.02*	89.83 ± 3.31^	78.83 ± 2.26*^$^
MDA (Mmol/g)		3.61 ± 0.28	6.05 ± 0.34*	4.03 ± 0.41^	6.1 ± 0.52*^$^

^a^Values are presented as mean ± SD.

Statistically significant (*p* < .05) as compared to the corresponding value in *group I, **^**group IIa and ^$^group IIb.

#### Maximum fused-tetanic tension without fatigue, after fatigue and with 5minrest following fatigue

When we compared maximum fused-tetanic tensions across the study groups; before and after rat muscles underwent fatigue and following a 5min-rest to recover from fatigue, positive control (untreated FM group) and GW9662-treated groups showed a statistically significant (*p* < .01) reduction in maximum fused-tetanic tensions compared to the negative control group. Noticeably, pioglitazone treatment resulted in improved muscle performance across the previously mentioned functions, which were comparable to those in the negative control group, i.e. no statistically significant differences were noticed (*p* > .05). It was clear from our data that the GW9662 treatment failed to show any improvement of muscle functions in FM rats and had similar results compared to the positive control group (*p* > .05), as shown in Table 1 and Chart 1.

### Biochemical parameters

#### Serum levels of IL-8 and MCP-1

**Chart 3.**
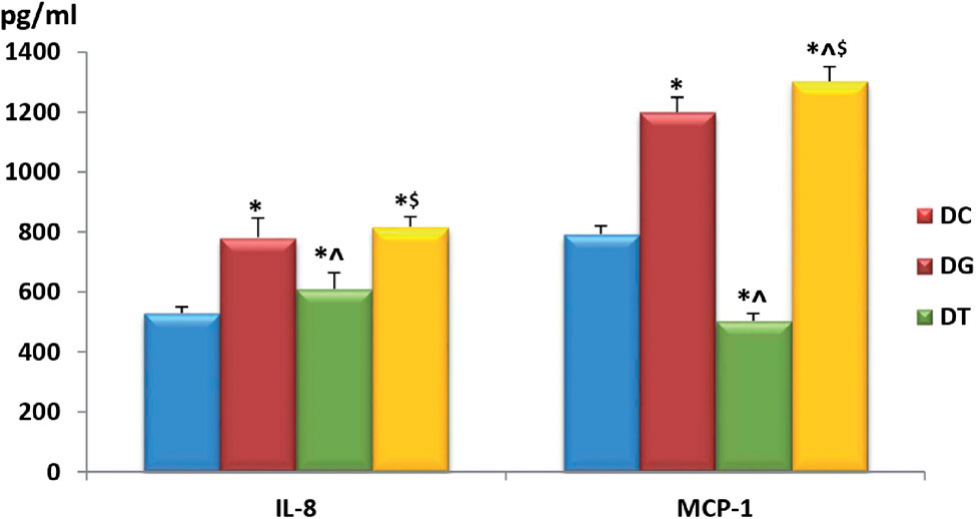
Comparison of serum IL-8 and MCP-1 levels among the study groups. Values are presented as mean ± SD. Statistically significant (*p* < .05) as compared to the corresponding value in *group I, **^**group IIa and ^$^group IIb.

As demonstrated in Table 1 and Chart 3, serum levels of both IL-8 and MCP-1 showed a statistically significant rise compared in FM rat models that were either untreated (positive control group) or treated with GW9662 (subgroup IIc) when compared to the negative control group (*p* < .01). There was no statistically significant difference in IL-8 levels between the negative control group and pioglitazone-treated rats (subgroup IIb). However, there was a noticeable, statistically significant reduction in MCP-1 levels in the latter subgroup (IIb) (*p* < .01) compared to the negative control group and rats in the other 2 FM-models (positive control and GW9662-treated groups).

#### Muscle homogenate levels of CAT, SOD and MDA

**Chart 4.**
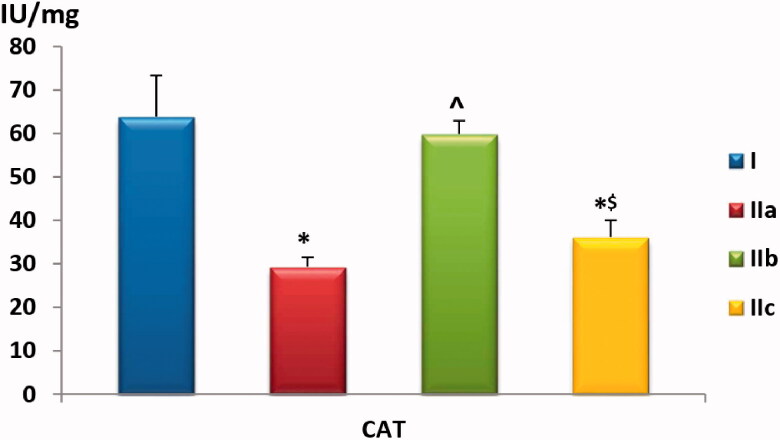
Comparison of muscle homogenate CAT level among the study groups. Values are presented as mean ± SD. Statistically significant (*p* < .05) as compared to the corresponding value in *group I, **^**group IIa and ^$^group IIb.

**Chart 5.**
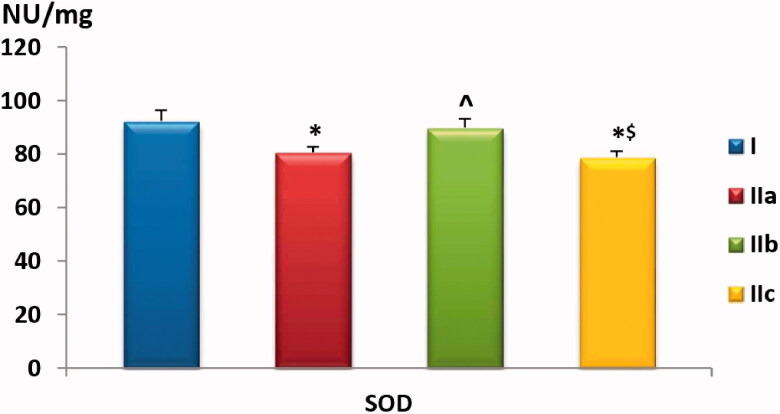
Comparison of muscle homogenate SOD level among the study groups. Values are presented as mean ± SD. Statistically significant (*p* < .05) as compared to the corresponding value in *group I, **^**group IIa and ^$^group IIb.

Our data showed a statistically significant (*p* < .01) reduction in CAT levels both in FM-untreated and GW9662-treated groups compared to the negative control group. However, Pioglitazone-treatment showed a protective effect against the loss of CAT activity with levels comparable to the negative control group (*p* > .05). There was a trend towards lower SOD activity in FM-control and the GW9662-treated groups compared to negative control or rats treated with Pioglitazone (*p* > .05), as shown in Table 1 and Charts 4 and 5.

**Chart 6.**
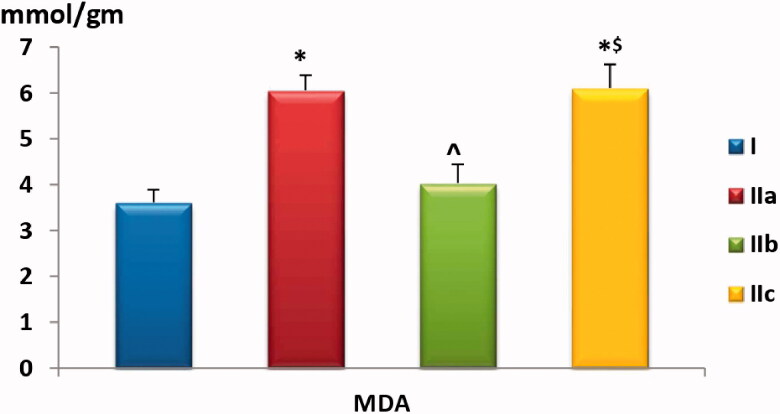
Comparison of muscle homogenate MDA level among the study groups. Values are presented as mean ± SD. Statistically significant (*p* < .05) as compared to the corresponding value in *group I, **^**group IIa and ^$^group IIb.

Last, we found a statistically significant (*p* < .01) increase in MDA levels among the positive control and the GW9662-treated groups compared to pioglitazone-treated rats, as shown in Table 1 and Chart 6

## Discussion

Fibromyalgia is a chronic debilitating disease characterized by widespread musculoskeletal aches, fatigue, sleep disturbances, among other somatic and cognitive manifestations. The manifestations usually last for many years and can sometimes cause significant impairment of quality of life. Despite the presence of many treatment regimens, effective management remains challenging [28].

Our goal in this study was to explore novel treatment options to control the musculoskeletal manifestations of FM adequately. Reserpine induces musculoskeletal deficits similar to FM, as shown by impaired motor activity of rat’s hind-limb muscles and significant reduction in antioxidant (SOD1 and CAT) activities. Skeletal muscle atrophy was recorded in the form of alteration of the muscle weight and diameter of myotubes. Reserpine also causes significant musculoskeletal ultrastructural alterations; besides the significant reduction of expression of endogenous antioxidants (SOD1 and CAT) and constitutive molecules involved in inflammation, oxidative stress and myogenesis processes (cyclo-oxygenase-1 (COX-1) and SIRT3) [29].

The level of cytokines varies in patients with FM. In a systematic review with meta-analysis, Üçeyler et al. [30] found that IL-6 is elevated in FM cases. This is supported by previous findings which linked FM-related changes to inflammation and oxidative stress [31,32]. Reserpine also caused a reduction in locomotor activity that had been related not only to long-lasting muscular mechanical hyperalgesia, tactile allodynia and tenderness [33] but also to depressive symptoms associating FM [16].

Treating FM rats with pioglitazone had a significant improvement of skeletal muscle functions, reduced fatigability and resulted in rapid recovery from fatigue. Blocking the PPAR-γ pathway, though administration of GW9662, counteracted pioglitazone protective effects. One possible mechanism underlying the protective effects of pioglitazone is the augmentation of PPAR -γ co-activator (PGC), which is highly expressed in skeletal muscle [34]. PGC-1 is one of the primary regulators of mitochondrial biogenesis, which acts by co-activating several transcription factors involved in mitochondrial DNA replication [35] and transcription [36] of genes encoding for mitochondrial β-oxidation enzymes [37,38]. PGC-1 also regulates skeletal muscle fibre type switching into oxidative slow-twitch fibres that are more adaptive to contraction-induced damage [39], glucose transport, and lipid utilisation [40]. It has been shown by others that loss of PGC-1α results in reduced muscle performance [41].

TZD’s have been tested for their anti-inflammatory effects on different mice and rat models. In one study, ciglitazone showed improvement of inflammatory process with peritonitis experimental sepsis rat model [42]. While pioglitazone improved adjuvant-induced arthritis (AIA) in rat models [43]. Also, pioglitazone protects against diabetic vascular complications independent from the decrease in blood sugar by decreasing atherosclerosis *via* different mechanisms [44]. Pioglitazone improved retinopathy in mice models through decreasing inflammation and neovascularisation in the retina in diabetic mice by decreasing TNF-alpha through stimulation of adiponectin, a known natural anti-inflammatory mediator [45].

The intracellular antioxidants SOD and CAT play a significant role in protecting cells against the damaging effects of ROS [46]. FM rats showed significantly elevated muscle levels of MDA, with decreased antioxidant activities of SOD and CAT when compared to normal healthy rats. Pioglitazone administration resulted in a significant rise in SOD and CAT levels together with a decrease in MDA levels, which were similar to levels normal healthy rats. Our findings come in agreement with a previous study using PPAR-γ agonists for the treatment of oxidative stress-related diseases [47].

PGC-1 is a positive regulator of endogenous antioxidant-enzymes expression [48]. Reduced mRNA and protein levels of SOD were observed in skeletal muscles of PGC-1 knockout mice, and those mice were more vulnerable to oxidative stress [49]. PGC-1 overexpression resulted in the up-regulation of skeletal muscle SOD levels [35]. In a PPAR-γ-dependent manner, the antioxidant enzyme CAT was induced, reducing oxidative stress and diminishing inflammatory response [50,51]. Besides, PPAR-γ protects from oxidative stress-induced apoptosis-inducing Bcl-2 [52]. This pro-survival action of PPAR-γ is probably independent of the mitogen-activated protein kinases (MAPKs)/Akt pathways [53]. Furthermore, ligand-activated PPAR-γ promotes the expression of manganese SOD (MnSOD), which oversees the dismutation of superoxide radical anion (O_2_^•−^) to O_2_ and H_2_O_2_ [54]. PGC-1 reduces mitochondrial ROS production by changing electron transport chain redox status [55], promoting mSIRT3 gene expression [56] that binds to, deacetylates, and activates mitochondrial enzymes, including SOD2, through a post-translational mechanism [57].

Intracellular lipids, including membrane phospholipids, reacts with O_2_^•−^ forming lipid peroxides that are metabolized to MDA and 4-hydroxynonenal [58]. Ligand-activated PPAR-γ induces the expression of scavenger receptor CD36 that mediates the recognition and internalisation of oxidized lipids [59].

Serum levels of the pro-inflammatory cytokines IL-8 and MCP-1 were significantly elevated in FM rats. Our results support what has been shown previously that increased release of inflammatory cytokines from circulating blood cells plays a role in the pathogenesis of FM [60–62]. These inflammatory cytokines attribute, to some degree, to FM key symptoms, such as fatigue, hyperalgesia and allodynia [63]. MCP-1 is a cytokine secreted by antigen-presenting cells. It plays a role in recruiting neutrophils and monocytes to sites of inflammation. The increased release of MCP-1 in the FM group explains, at least in part, reduced levels of regulated upon activation, normal T cell expressed and presumably secreted (RANTES) by the monocytes [64]. Although the results of previous studies investigating systemic levels of MCP-1 in MF have been controversial [65,66], there was a belief that the release of RANTES by monocytes is altered in FM. The results presented here confirm the hypothesis of a dysregulated inflammatory response in FM that involves monocytes. Our results showed a significant reduction in serum levels of IL-8 and MCP-1in pioglitazone-treated rats to an extent similar to that of normal healthy counterparts.

## Conclusion

Pioglitazone, the PPAR-γ agonist, emerges as a promising cure to restore and promote skeletal muscle functions and alleviate fatigue associated with FM syndrome. A possible mechanism is through restoring pro-oxidant/anti-oxidation balance.

## Data Availability

Data are available on request from the corresponding author.
