# Correction

**DOI:** 10.1080/07853890.2023.2273659

**Published:** 2023-10-26

**Article title:** Pioglitazone improves skeletal muscle functions in reserpine-induced fibromyalgia rat model

**Authors:** Hassan, F. E., Sakr, H. I., Mohie, P. M., Suliman, H. S., Mohamed, A. S., Attiaf, M. H., & Eid, D. M.

**Journal:** *Annals of Medicine*

**Bibliometrics:** Volume 53, Number 01, pages 1033–1041

**DOI:** 10.1080/07853890.2021.1916069

When this article was first published online, the Ethical Committee approval number in first paragraph of the *Materials and methods* section was incorrectly listed as CU/III/F/68/18.

This has now been revised and the sentence updated with the correct ethical committee approval number, as follows: “The described protocols in this study were approved by the Institutional Animal Care and Use Committee (CUIACUC), Cairo University, with the approval number: CU/I/F/66/20.”

